# University of Maryland, Dental Department

**Published:** 1891-04

**Authors:** 


UNIVERSITY OF MARYLAND, DENTAL DEPARTMENT.—The Annual Commencement exercises of the Department of Dental Surgery of the University of Maryland for 1891 were held at the Academy of Music, Baltimore, Md., on Wednesday, March 18th.

The exercises opened with prayer by the Rev. Dr. A. C. Tuttle, of Mount Vernon M. E. Church. The mandamus was read by the Dean, Prof. Ferdinand J. S. Gorgas, M. D., D. D. S.; the address to the graduates was delivered by Rev. J. J. G. Webster, D. D., of Madison Avenue M. E. Church, and the class oration by William H. Conner, D. D. S., of New Jersey.

The number of graduates was sixty-four, and the number of matriculates for the session was one hundred and sixty-three.

The degree of D. D. S. was conferred on the following graduates by the Dean, Professor Gorgas, in the absence from illness of Hon. S. Teackle Wallis, LL. D., Provost of the University:

Harry W. Allwine, Nebraska; J. Perrin Anderson, South Carolina; Henry C. Bagby, M. D., California; Denison Holmes Baldwin, Virginia; Frederick A. Barr, Montana; James G. Benjamin, Montana; Capers W. Blalock, Florida; John D. Booth, Canada; J. William Boozer, A. B., South Carolina; Aubrey R. Bowles, Virginia; Emory M. Bowlus, Maryland; Charles S. Boyette, North Carolina; Elvie S. Boyle, Maryland; Samuel S. Brotherton, Iowa; George L. Bruce, Maryland; F. Wayne Chessrown, Pennsylvania; M. H. Pettway Clark, North Carolina; William H. Conner, New Jersey; Geo. L. Deichmann, Maryland; William Earle, South Carolina; Will H. Ewald, Virginia; William Meade Feild, Virginia; Herbert F. Gorgas, Maryland; J. William Grove, Pennsylvania; W. Oakley Haines, Maryland; Jacob V. Haller, Virginia; Henry F. Harris, Virginia; Marion Y. Hart, Virginia; Will W. Hayes, Pennsylvania; J. Henry Hoffman, Maryland; Charles W. Howard, New Hampshire; Edward T. Jones, Virginia; John C. Lœschcke, Virginia; Edwin A. Martin, L. D. S., Canada; Oscar Matt, New York; Virgil J. McComb, Missouri; Clyde M. McKelvey, Pennsylvania; T. Benton Moore, Pennsylvania; Frank A. Pattison, New York; William B. Poist, District of Columbia; William E. Prather, North Carolina; Bernard F. Riedel, Maryland; Richie W. Riley, South Carolina; Johannes Rilke, Germany; Frank Robinson, L. D. S., England; George B. Rounds, Canada; Heinrich Sengebusch, Germany; Henry B. Snow, Maryland; E. Alva Solomons, South Carolina; Alfred E. Sparks, Canada; A. Hume Sprinkel, Virginia; C. Carfer Sprinkel, Virginia; F. Myron St. John, Connecticut; Frazer P. Stehley, West Virginia; J. Frank Stevens, Pennsylvania; George W. Stevenson, New York; R. E. Lee Taliaferro, Virginia; Charles A. Turner, Canada; Adolf W. A. Volck, Germany; Albert S. Wells, North Carolina; George F. White. New York; Robert J. Whitfield, Canada; Charles E. Wingo, M. D., Maryland; Charles Rockwell Wood, New Hampshire,

The University prize, gold medal, was awarded to Aubrey R. Bowles, of Virginia, for the highest number of votes at the final examinations. Virgil J. McComb, D. D. S., of Missouri, received honorable mention for the second highest number of votes.

The Alumni Banquet was held after the Commencement exercises.

